# Circulating tumour DNA monitoring and early treatment for relapse: views from patients with early-stage melanoma

**DOI:** 10.1038/s41416-022-01766-x

**Published:** 2022-03-17

**Authors:** Victoria G. Woof, Rebecca J. Lee, Paul Lorigan, David P. French

**Affiliations:** 1 Manchester Centre of Health Psychology, Division of Psychology & Mental Health, School of Health Sciences, Faculty of Biology, Medicine and Health, University of Manchester, Oxford Road, Manchester, M13 9PL UK; 2 The Christie NHS Foundation Trust, Wilmslow Road, M20 4BX Manchester, UK; 3 Faculty of Biology Medicine and Health, The University of Manchester, Oxford Road, M13 9PL Manchester, UK

**Keywords:** Melanoma, Skin manifestations, Psychology

## Abstract

**Background:**

Circulating tumour DNA (ctDNA) monitoring is a new technology that detects cancer DNA fragments in blood circulation. Regular monitoring with ctDNA has the potential to detect and treat cancer relapse earlier, but there is little evidence on patient acceptability. This study examines the views of patients with early-stage melanoma on the acceptability of the test and early treatment.

**Methods:**

A qualitative cross-sectional design using one-to-one semi-structured telephone interviews was employed. Twenty-five patients diagnosed with early-stage melanoma (Stage IA–IIC) were asked for their views on ctDNA monitoring and early treatment for relapse. Interviews were analysed using reflexive thematic analysis.

**Results:**

Two themes were generated: *ctDNA monitoring would add service value* where participants described regular ctDNA monitoring in follow-up care as more reassuring, more “scientific” than skin checks and preferable to scans. *Test results provide opportunity and knowledge* focuses on how participants wanted to know when to expect results to manage anxiety, with a positive result seen as an opportunity to receive treatment early.

**Conclusions:**

Participants were positive about ctDNA monitoring and early treatment and would welcome extra surveillance, as well as trust ctDNA tests. This indicates the feasibility of the wider implementation of ctDNA tests, which have applicability for many tumour types and disease stages.

## Introduction

Biomarkers are commonly used to assess the patient’s prognosis, indicate the presence or activity of cancer, or to predict treatment response. Whilst many of these rely on tissue biopsies, blood-borne biomarkers have the advantage of being readily accessible and can be measured serially. A promising new blood-borne biomarker is circulating tumour deoxyribonucleic acid (ctDNA), which is DNA present in the bloodstream arising from cancer cells that can be detected through identifying somatic mutations in plasma. Over the last few years, numerous studies have demonstrated its utility as a prognostic biomarker, a predictive biomarker of response to different therapies and a tool to characterise mechanisms of resistance to treatment [1–5]. More recently, studies have shown that it can be used to identify the minimal residual disease and molecular relapse following curative-intent surgery/treatment for early-stage cancers [6–8].

One potential application of ctDNA is the detection of early relapse following curative-intent surgery or (neo)adjuvant therapy for early-stage melanoma [9, 10]. The individual risk of recurrence for these patients is low, however, because it is more common to be diagnosed with early-stage melanoma, as a group they contribute to 30–50% of all melanoma deaths [11]. Given this challenge, the DETECTION trial (Circulating tumour DNA guidEd Therapy for stage IIB/C mElanoma after surgiCal resection; ClinicalTrials.gov identifier: NCT04901988) is underway to assess whether early treatment based on detection of ctDNA in patients with resected stage IIB/C melanoma improves survival [12]. A key determinant of the success of this strategy is whether it is more acceptable than standard care for this group, which is currently regular clinical examination (skin checks and palpation of lymph node basins) with cross-sectional imaging.

Due to the relative novelty of ctDNA being used in clinical decision-making, there is a paucity of data regarding patient acceptability of this approach and whether regular testing could result in anxiety. We, therefore, used qualitative methods to investigate the acceptability of ctDNA blood test monitoring and early treatment for relapse based on ctDNA test results from the perspective of patients with early-stage melanoma (Stages IA–IIC). Patient acceptability in this study is defined as how palatable or satisfactory ctDNA monitoring is with regards to the complexity of the intervention as well as the procedures being used [13]. Findings from this study will inform the implementation of ctDNA monitoring in the DETECTION trial and potentially in future clinical practice. It will also provide insight into the wider acceptability of this new technology to cancer patients with other tumour types where trials are in setup or ongoing, including breast [14] and colon [15] cancers.

## Methods

### Design

This study employed a qualitative cross-sectional design using one-to-one telephone interviews. The consolidated criteria for reporting qualitative research (COREQ) checklist was used in the reporting of this study [16] (Supplementary Material 1).

### Participants

Patients were eligible to participate if they were aged 18 years or over and had ever been diagnosed with early-stage melanoma (Stage IA–IIC), without locoregional spread or distant metastases. Participants were required to communicate in the English language for the interviews.

### Procedure

Prior to the COVID-19 pandemic, recruitment was proposed to be via dermatology clinics at a North West England NHS Trust. However, as the majority of this study was conducted during the height of the UK pandemic this form of recruitment was not possible, due to patient access and limited NHS staff capacity. Instead, it was decided that participants were to be recruited using an advertisement displayed on social media, which tagged UK melanoma charities, Melanoma Focus UK and Melanoma UK. The advertisement and information materials were also sent via email to charity representatives at Melanoma Focus UK and Melanoma UK who distributed the materials to their supporters and posted information on closed melanoma support groups on Facebook. Those interested in participating were required to contact the researchers for more information.

Interviews were semi-structured to enable an in-depth insight into the views of patients with early-stage melanoma on ctDNA monitoring and early treatment for molecular relapse, with the use of probes to explore particular areas of interest. A public, patient involvement and engagement (PPIE) group helped inform the topic guide and suggested that a definition of ctDNA be provided if participants did not have any prior knowledge of ctDNA and the blood test procedure.

To ensure a level of neutrality, members of the research team directly involved with the DETECTION trial did not carry out the interviews. Before each interview began, all participants provided verbal consent to take part, which was audio recorded separately from interview data. Participants were initially asked if they were familiar with the term ctDNA and those who were not were provided with a simple definition to help them answer questions, as advised by the PPIE panel. During the interviews, participants were asked about their thoughts on a blood test for ctDNA, which could indicate early signs of relapse and about receiving treatment early if ctDNA was detected. Interviews lasted 30–60 min and were conducted by VGW, an experienced researcher in qualitative research methods.

### Analysis

Data were analysed in NVivo12 using reflexive thematic analysis [17]. An essentialist perspective informed the analysis, with the researchers adopting an inductive-semantic approach to coding. This form of analysis was chosen as the researchers aimed to interpret the data free from (as far as is possible) the influence of pre-existing ctDNA literature. To ensure this neutrality, the researcher conducting the analysis had minimal background knowledge of melanoma, ctDNA and the DETECTION trial. The essentialist perspective allowed the researchers to highlight and interpret the participants’ assumed realities within the data. Initial coding was completed by VGW. Coding was iterative, with codes and patterns compared and continually refined as more data were analysed. Related codes were then grouped together in order to form descriptive patterns of the data. During this process, initial themes were developed as patterns within the codes became more refined. Codes and themes were refined and discrepancies discussed at coding meetings between VGW, DPF and RJL before the final thematic structure was deemed representative of the sample. Data sufficiency was achieved and recruitment stopped when the researchers believed that, (1) no new information was being discussed in the final two interviews, (2) that new avenues of interest which had spontaneously arisen in interviews had been thoroughly explored in subsequent interviews and (3) that there was sufficient data to answer the research question and study aims [18].

## Results

### Sample demographics

Twenty-five participants from across the UK were interviewed who were diagnosed with early-stage melanoma (Stages IA–IIC) between 2011 and 2021 (see Table 1 for a description of the cohort).

**Table 1:** Demographic and clinical characteristics of the participating melanoma patients.

Characteristics	Sample (*n* = 25)
Age, years	
Median (min, max)	48 (27.0, 66.0)
Interquartile range (IQR)	41.0–53.0
Gender, *n* (%)	
Female	21 (84)
Male	4 (16)
Melanoma stage, *n* (%)	
Stage IA	11 (44)
Stage IB	6 (24)
Stage IIA	5 (20)
Stage IIB	2 (8)
Stage IIC	1 (4)
Year of diagnosis, *n* (%)	
Pre 2015	6 (24)
Post 2015	19 (76)
Born in the UK, *n* (%)	
Yes	22 (88)
No	3 (12)
Ethnicity, *n* (%)	
White—British	22 (88)
White—Other	3 (12)
First language spoken, *n* (%)	
English	24 (96)
Spanish	1 (4)
Education, *n* (%)	
O’ levels/GSCE	3 (12)
A levels/other post-16 qualifications at college	7 (28)
Degree	7 (28)
Post-graduate qualification, e.g. masters, PhD	8 (32)
Index of Multiple Deprivation (IMD) score	
Median (min, max)	6 (1, 10)
IQR	4–8

### Findings

Views from participants regarding ctDNA monitoring for signs of early relapse and early treatment were grouped into two themes: (1) ctDNA monitoring would add service value and (2) test results provide opportunity and knowledge, with each theme including subthemes. All participant names have been replaced with pseudonyms.

### Theme 1—ctDNA monitoring would add service value

The majority of participants did not know what ctDNA monitoring was or were unable to provide a definition. Upon providing participants with a simple definition of the new technology and blood test procedure, all of the participants described regular ctDNA monitoring as a good idea, as the new technology would be more ‘scientific’, would reduce the fear of the unknown and identify relapse early.

#### Subtheme 1—ctDNA monitoring is a more systematic approach to follow-up care

The majority of participants described skin check procedures as inconsistent in quality. They explained that at each appointment checks would be performed by a different healthcare professional, with some more thorough than others. They felt that skin checks are subject to human error and feared that changes could be missed. Consequently, participants believed that more could be done to provide them with a consistent marker of relapse:

*I’ve gone from having really detailed full body checks, lymph node checks, et cetera, every single one of the moles – and I have a lot of them on my body – being checked, to, okay, we’ll check your lymph nodes, we’ll look at the original site, are there any that you’re worried about, type thing? (Fiona, 42, stage 1b, diagnosed in 2015)*

When presented with the idea of a blood test to detect for early signs of relapse, all participants reacted positively, identifying ctDNA monitoring as the next step in melanoma care:

*I think the treatment, as they stand at the moment, is all pretty visual. You know, on the whole, especially for stage 1 and 2, I think it’s virtually all visual, and things can get missed. Whereas, if you’ve got definite results, or likely results, from something like a blood test, I believe that to be more the way forward. (Harry, 49, stage 2b, diagnosed in 2017)*.

Compared to skin checks, they described a blood test as a more ‘scientific’ measure, providing conclusive evidence of cancer recurrence. They explained that if ctDNA monitoring had been offered as part of their follow-up care, they would have been more reassured, describing the test as an extra safety measure or as one participant described ‘another line of defence’ (Graeme, 34, stage 2a, diagnosed in 2021):

*I’d be over the moon if someone said to me, we’re going to put you on regular checks for x number of years as well as these visual skin checks. It feels more scientific. (Gill, 51, stage 1a, diagnosed in 2019)*

As well as mitigating the effects of human error at skin checks, participants explained that blood test monitoring for early signs of relapse would be essential for providing internal evidence of change. Nevertheless, although deemed more conclusive than skin checks, all participants believed that ctDNA monitoring should not replace visual checks but be used alongside them for a ‘belt and braces’ approach to care:

*I just think it’s like a double check really isn’t it, it’s like a belt and braces, you’ve got a visual and then you’ve got the internal and you’ve got something scientific that can be kind of highlighting you as early as possible. Especially if it’s being done every three months, that’s so quick in terms of, if you start to see something then, you could do something quite preventable. (Yvonne, 47, stage 1b, diagnosed in 2019)*

#### Subtheme 2—ctDNA monitoring would reduce patient anxiety and increase reassurance

Participants described melanoma as a ‘sneaky’ disease with no reliable means of knowing whether relapse would occur. This fear of the unknown was identified as anxiety-provoking and hard to live with post-diagnosis. However, the participants explained that a regular blood test would provide them with the confidence and peace of mind that they were being monitored for melanoma relapse more closely:

*…well certainly me with melanoma, you know, that’s absolutely my biggest fear, is that it’s going to come back and I’m not going to know about it, I’m not going to know about it until, you know, I’m so much further down the line so to speak. So, you know, if anything could detect something early and there could be early intervention then, you know, I would want to have it. (Lisa, 32, stage 2a, diagnosed in 2018)*

They suggested that opting for this blood test would enable patients to feel more proactive about their care, reducing anxiety and enabling them to be forewarned about a potential relapse. Similarly, all participants felt that with regular blood tests, the anxieties regarding small changes to the skin and moles that occur between appointments could be better managed due to perceived additional surveillance:

*…say you’re 1A and then finding a lymph node enlarged. That might happen in a second because you might find it and your mind does this massive jump, whereas if you were being monitored you’d have a bit of logic. You’d be able to think well, I was monitored however long ago, this could be something else. I think it would just add a kind of reassurance that more than you poking and prodding your own body around. (Louise, 47, stage 1a, diagnosed in 2019)*

#### Subtheme 3—ctDNA monitoring would be a valuable early detection measure

The majority of participants viewed having regular blood tests for ctDNA monitoring could be useful as an early detection measure for relapse. Although the thought of relapse was difficult to consider, participants felt that a ctDNA test would provide them with the best opportunity to catch recurrence early before presentation with symptoms or radiological detection:

*…if it can detect something that might…that wouldn’t necessarily get picked up because obviously melanoma travels in the blood and unless you’ve got something, I don’t know, maybe a mole or a lump, or something like that, you would never know until that. But obviously if this could pick that up beforehand then I think it would be a good thing. (Rebecca, 49, stage 1a, diagnosed in 2015)*

In addition due to the radiation risks associated with computerising tomography (CT) scans, regular blood tests were deemed more favourable for some participants:

*I think a blood test would be better than a scan, in levels of progression you would get your skin check, your blood check and then a scan would be the next step after that I’d say. So it saves you having to have unnecessary exposure to radiation and things like that, which obviously is another cause of cancers. (Cathy, 34, stage 1a, diagnosed in 2019)*

However, not all participants agreed that early treatment following the detection of ctDNA was appropriate, as a minority questioned whether treatment should be provided only after evidence of a tumour has been found via a CT scan, as they believed treatment prior to the identification of mass could be unnecessary:

*It’s hard, isn’t it, because I think if you’ve actually got cancer and it’s there and you know it’s there, you want to do everything you can to get rid of it, don’t you? […] But if it’s not actually come yet, do you really want treatment that could make you ill? (Jean, 63, stage 1a, diagnosed in 2017)*

#### Subtheme 4—regular ctDNA monitoring is desirable

For the reassurance it would provide a number of participants explained that they would be happy to receive a ctDNA test for the rest of their lives, especially if the frequency of the blood test was manageable:

*So if it was every few months, every three months or longer then I’d probably be happy for it to go on for longer or indefinitely if need be. (Eric, 39, Stage 2a, diagnosed in 2018)*

However, they understood that this might not be possible due to NHS funding, as well as the scientific rationale that the risk of recurrence decreases over time. For ctDNA monitoring to add the most value to patients, the majority explained that monitoring should be more frequent closer to diagnosis and reduced as the risk decreases, identifying a need for a risk-stratified approach:

*I suppose it would depend how far after your diagnosis you were and what your dermatologist said were your chances of it recurring. So possibly the same as skin checks where it’s more frequent in the beginning and maybe spaces out as you get further away from it. (Louise, 47, stage 1a, diagnosed in 2019)*

They described that three monthly for three years and 6 monthly for 2 years would be ideal as it would fit in with the existing care structure for stage two patients in the UK. Despite this ideal timeline, all participants stressed the importance of identifying ctDNA in the blood at the earliest opportunity. They, therefore, explained that if ctDNA can be identified earlier or later than three months then the frequency of blood tests should reflect this:

*So it would depend on, I guess, the evidence for how likely you would expect to see changes. So if it’s once a year, six months, three months, I would say yes to whatever people gave me because it sounds like a preventable strategy kind of thing. (Yvonne, 47, stage 1b, diagnosed in 2019)*

### Theme 2—test results provide opportunity and knowledge

All participants described waiting and receiving test results as anxiety-provoking regardless of the outcome. They felt that being informed as to when to expect their ctDNA test results would improve anxiety. Notification of a negative test result would provide them with the reassurance to move on with life between tests. A positive test result, although worrying was viewed as an opportunity to access treatment early and receive better health outcomes.

#### Subtheme 1—providing a timeline for results is helpful

All participants described waiting for test results as an anxious time and a result for a ctDNA blood test would be no exception. Participants identified that until results were received, there is constant speculation about the outcome, with some not being able to relax during the waiting period:

*…once you have a test for something you’ve always got it in the back of your mind until the results come in, so I wouldn’t be totally on edge all the time, but it would be in [the] back of my head all the time. (Vanessa, 57, stage 2b, diagnosed in 2018)*

With result anxiety in mind the majority of participants cited under two weeks as an appropriate time to receive notification of results. Others explained that waiting for their blood test result would depend on laboratory turnaround times in processing blood samples. However, no matter how long results would take, all participants explained that they would require notification of when to expect their results enabling them to manage their anxiety:

*…if they don’t know what timeframe it’s going to be expected in, some people’s anxiety levels may be really, really high, straight from the off […] Whereas, if they know it’s not going to be expected for two weeks, a month, then they can at least get on with their lives, and not have it right at the forefront of their thoughts all the time. (Sam, 53, stage 1b, diagnosed in 2015)*.

Yet some identified that after attending for multiple blood tests receiving results would become routine and less worrying over time:

*I don’t think I would be particularly thinking about results coming through. I can imagine I’d forget about it really over time and a letter or whatever it is would arrive every two months and I wouldn’t be anticipating receiving it or anything like that. (Eric, 39, stage 2a, diagnosed in 2018)*.

#### Subtheme 2—notification of a negative ctDNA result essential

When they considered how it would feel to receive ctDNA results, all participants said that they would be happy and trust their result if it were negative. All participants explained that they would want to be notified of a negative test result, as lack of notification would be unacceptable. They argued that they would not accept ‘no news is good news’ but would instead worry and speculate about their result, with some being prepared to call services directly for confirmation:

*I think, if I wasn’t notified, I’d be wondering if the letter is stuck in the post or if there’s some problem at the hospital notifying, or there was an admin error. To have a negative result is much better than to be told if you’re positive because it stops you worrying - I just want to check that that was okay and it’s just that you haven’t sent it out or I think I, personally, would still ring up about a few weeks later to say just want to check what results are on the file or I’d bother my GP for it. (Gill, 51, stage 1a, diagnosed in 2019.)*

Participants explained that a simple letter or text message notifying them of a negative result would provide them with peace of mind before their next appointment. Knowledge of a negative test result for ctDNA was viewed as something that could enable patients to move on with their lives between tests, providing them with time to enjoy life and worry less. Specifically, one participant explained that consistent notifications of negative test results would improve their state of mind regarding a potential relapse:

*I think as well, the more times that happened [receiving a negative test result], so the further into those five years I got, I would become more and more reassured. You know, at three years, at four years, I’d start to be probably quite optimistic. Because, again, I know that most melanoma reoccurs within the first two years. And I know that if you get to five years, you know, it’s quite a… It’s a very positive milestone (Graeme, 34, stage 2a, 2021)*

However for a minority, the thought of ‘there is always next time’ would be difficult to ignore, meaning any reassurance gained from a negative test result would be temporary. For example, one participant explained that the level of reassurance a negative test result would provide would depend on when the test was taken during their follow-up, with a negative ctDNA result at the beginning of monitoring being less significant than one received towards the end of follow-up:

*I think it depends how long after you’ve been diagnosed that the test takes place, you know, where you are on your long term journey I suppose. If it’s, you know, a few months after I think you’d be waiting for the next test for something, you know, you’d be expecting something else to be happening next time in terms of spread. (Steph, 52, stage 2a, diagnosed in 2018)*

#### Subtheme 3—a positive ctDNA result is an opportunity

Participants explained that a positive ctDNA blood test result would provide them with a chance to be treated early. Although a positive test result is not good news, they would be grateful that relapse was identified before cancer had time to progress and present visually:

*Well, obviously that’s [a positive result] going to cause a bit more anxiety, isn’t it? But also at the same time there could be a bit of a relief there, you know, that it’s actually been caught rather than it not being picked up. So it can work either way, really. For me, if it was positive, I’d rather know. That there’s something going on, and maybe we can do something about it. (Jean, 63, stage 1a, diagnosed in 2017)*

The majority of participants explained that they would soon deal with the initial shock of a positive test result and instead would concentrate their minds on what needed to be done to reduce the chances of cancer progressing:

*Well, the first word that came to my mind was devastated, gutted. But, I’d be like, right, okay, it’s been caught, before it can hopefully get anywhere, like if you’re in regular testing? I’d think, right, it’s been caught early, what are we going to do about it? (Cathy, 34, stage 1a, diagnosed in 2019)*

As the majority would want to immediately know their management plan, they suggested that information material, such as leaflets and result letters state clearly what a positive test result would mean. Should a positive test result be delivered by letter, participants stressed the importance of having the opportunity to speak with a healthcare professional or have an appointment automatically arranged. Others believed that a positive test result and notification of needing treatment should be delivered in person to enable the patient to ask questions and be provided with the space and time they need to process the information with a healthcare professional present.

## Discussion

The patients interviewed were positive about regular ctDNA monitoring for melanoma recurrence, describing the test as more ‘scientific’ and more desirable than physical examinations. Patients viewed the test as a way of providing reassurance, they trusted it and thought it enabled early relapse detection, with early treatment potentially preventing cancer progression. To manage anxiety, it was deemed vital to know when to expect notification of results, with notification of a negative result essential to enable patients to enjoy life between appointments. In addition, patients would want to be given the results in a timely manner with a maximum 2-week wait. Although daunting, a positive result would provide an opportunity to be treated early, and in the patients’ opinion, lead to better health outcomes.

The use of ctDNA for relapse monitoring and treatment decisions is currently being tested in clinical trials and thus, as it is a new technology, little is known about its acceptability to patients. The findings presented here highlight the positive views that patients have towards implementing this new form of monitoring as part of their follow-up care. In fact, their views may be overly positive, due to the perceived ‘scientific’ nature of ctDNA compared to the uncertain clinical utility of the assay [12]. Positive views toward ctDNA monitoring for relapse have also been found amongst women diagnosed with endometrial cancer [19].

Previous research indicates that patients with cutaneous melanoma generally view their follow-up care (including skin checks) as reassuring and an effective way to monitor cancer recurrence [20]. This is somewhat at odds with the findings of the present study as participants described skin checks as a variable service dependent on the type of healthcare professional conducting the appointment. Instead, this study has shown that ctDNA monitoring, together with skin checks would enhance follow-up care and increase patient reassurance. Women diagnosed with endometrial cancer have also reacted positively to a ctDNA blood test compared to the current standard of care practices, with a blood test preferred to a pelvic exam [19]. It should be noted that not all participants in the present study were convinced of cancer being present if it was undetectable via a scan, and hence were uncertain as to whether treatment would be necessary. This finding that patients prefer to monitor disease using palpable signs and symptoms is in line with previous findings. For example, some women with breast cancer self-monitored for cancer progression during tamoxifen therapy by feeling lymph nodes and breast self-examination, with some women becoming anxious when their lymph nodes shrank as it did not allow such monitoring [21]. However, in another study, women diagnosed with endometrial cancer expressed a desire to know their ctDNA blood test results even if the recurrence could not be detected via a scan, although one woman placed more faith in a CT scan for detecting cancer relapse [19]. It will be important to explain to patients the evidence for the association of ctDNA detection with relapse so they can better understand the efficacy of the test.

Waiting for cancer test results causes stress and anxiety [22], especially when the waiting period is particularly long [23, 24]. Our study supports procedures whereby negative test results are reported quickly to enable patients to experience closure from their tests. Participants in the present study also expressed a desire to have an active role in their care. They explained that they would want to be explicitly informed about ctDNA monitoring and subsequent treatments if ctDNA was detected. They indicated a need for written information materials and the opportunity to discuss with healthcare professionals, which is consistent with previous findings in relation to melanoma cancer care [25]. Following a diagnosis of cancer, patients can experience a loss of control over their life and care [26], therefore providing ctDNA monitoring results and a clear action plan following a positive result could potentially be empowering.

As the ability of this technology to improve patient outcomes is still being evaluated, it was not possible to interview patients who are receiving these tests as part of routine healthcare. Acceptability is a dynamic concept, thus patients’ views regarding the acceptability of ctDNA are likely to differ from pre-implementation/conception to when the procedure is actually experienced in practice [13]. Future research will prospectively explore views and anxiety levels of those receiving ctDNA monitoring in clinical settings. It will be important to understand how those people who receive positive test results from ctDNA testing react to this diagnosis, relative to patients who receive positive test results from more traditional routes. Therefore, it will be important to perform future research evaluating patients who are undergoing ctDNA testing. In addition, due to the novelty of the test, participants in the present study either did not know or had a limited understanding of what ctDNA was, therefore the researcher provided each participant with a lay definition of ctDNA. Thus, this simple and jargon-free definition could have influenced participants’ views on the acceptability of the test and their positive attitudes towards ctDNA. To reduce the potential for bias we received input from a PPIE group regarding a neutral, understandable definition.

Recruitment for this study was originally designed to be via a NHS dermatology clinic. Due to the COVID-19 pandemic, recruitment was forced online, with adverts placed on Twitter, closed Facebook groups and via UK melanoma charity mailing lists. This meant that the sample obtained over-represented highly educated younger women and hence may not have captured the views of other groups (i.e. less educated people and men). However, when comparing the views of both the male and female participants in the sample, there appeared to be no distinct differences in how either of the sexes viewed ctDNA monitoring for early relapse. Furthermore, current UK cancer statistics indicate that melanoma is most prevalent in adults over the age of 70 and less prevalent in younger adults [27]. In this study, the eldest participants were in their late 60s. Therefore, according to UK statistics, this cohort of melanoma patients could be considered as unrepresentative of a typical patient over the age of 70. However, it should be noted that opinions did not vary between the younger and older participants in this sample. Furthermore, it is important to highlight that with the increased use of sunbeds, melanoma is more common among younger adults than any other cancer [27]. Nevertheless, future work examining patients undergoing ctDNA testing on the DETECTION trial will in a larger sample size reducing this potential bias.

In this study, ctDNA testing was viewed as a useful additional procedure that would enhance follow-up care for melanoma patients, rather than be a replacement for regular skin checks. As previously discussed, not all participants were convinced of cancer being present if undetectable via a scan, causing uncertainty as to whether treatment is necessary. Should clinical trials show a benefit for ctDNA monitoring, it may be useful for clinicians to provide evidence to inform patients of the degree of certainty surrounding a ctDNA result. Findings to date indicate that ctDNA detection is highly specific for identifying early relapse, with figures of 95–100% specificity being identified within 12 weeks of surgery [9, 10]. If this specificity could be communicated effectively, ctDNA monitoring via a blood test was viewed as more favourable than CT scans due to its associated risks of radiation, and so may be intrinsically more acceptable. In either case, a number of patients felt that ctDNA was more ‘scientific’ and reliable than skin assessments so it is critical to highlight to patients that they should continue to perform self-examination, as ctDNA is less likely to identify small volume local recurrence/new primaries compared to distant/internal organ metastases [28]. Further qualitative work should investigate how best to convey the accuracy of ctDNA testing in order to establish trust in its results, especially for those patients who would rather see a tumour on a scan than rely on a blood test.

For all participants, notification of when to expect their ctDNA results would enable them to manage their anxiety. This feeling has been echoed in research in patients with endometrial cancer [19]. All results need to be communicated, as no notification of a negative test result would not be accepted. On receipt of a positive test result, timely information with access to a healthcare professional will be key. These findings mirror that of patients receiving notification of skin biopsies, where rapid communication of results and access to a healthcare professional to manage patient queries was deemed important [29]. It is critical therefore that cancer services and healthcare professionals explain the consequences of a positive test result prior to patients embarking on ctDNA monitoring and repeat this information following a positive test result, as this could be after a long period of time has elapsed. This would enable patients to feel informed and empowered about their follow-up care and treatment pathway.

For some, having ctDNA monitoring for the duration of their lives was desirable. Expectations will need to be managed with regards to how long and how often ctDNA monitoring is required. A rationale behind the monitoring frequency will need to be communicated to reassure patients that the threat of relapse is less as monitoring is brought to an end. Future research should aim to assess patient anxiety levels regarding relapse following the completion of ctDNA monitoring once it has been clearly established as to when it is safe to stop.

Overall, participants with early-stage melanoma were very positive regarding ctDNA monitoring for relapse as part of follow-up care following curative-intent treatment. This test would be trusted and would reassure patients that relapse was being monitored closely, enhancing existing follow-up procedures and creating a more systematic approach to care. Given the potential for widespread application of ctDNA testing in many tumour types, this study provides important insights into the practicalities of its delivery and its value for patients.

### Reporting summary

Further information on research design is available in the Nature Research Reporting Summary linked to this article.

## Supplementary information

COREQ checklist

Reporting summary

## Data Availability

The dataset used in this study is not publicly available as it may contain information that would compromise participant consent. Please contact the corresponding author for more information.
